# Matrix-producing Breast Carcinoma: A Rare Subtype of Metaplastic Breast Carcinoma

**DOI:** 10.7759/cureus.5188

**Published:** 2019-07-22

**Authors:** Nektarios Koufopoulos, Stefania Kokkali, Foteini Antoniadou, Dionysios T Dimas, Ioannis L Missitzis

**Affiliations:** 1 Pathology, Attikon University Hospital, Medical School of Athens, Athens, GRC; 2 Oncology, Saint Savvas Cancer Hospital, Athens, GRC; 3 Pathology, Saint Savvas Cancer Hospital, Athens, GRC; 4 Surgery, Saint Savvas Cancer Hospital, Athens, GRC

**Keywords:** matrix producing carcinoma, metaplastic breast carcinoma, local recurrence, distant metastasis, breast tumor

## Abstract

Matrix-producing carcinoma (MPC) is a rare subtype of metaplastic breast carcinoma (MBC) that was first described in 1989 by Wargotz and Norris. It accounts for less than 1% of breast carcinomas and has distinctive clinical, morphological, and immunohistochemical features. Histologically it consists of invasive carcinoma of no special type with transition to cartilaginous or osseous matrix without a spindle cell component. Data on this entity are limited with the literature consisting mostly of case reports and a small number of case series.

We report a case of matrix-producing breast carcinoma, with excellent clinical outcome. We also discuss the histogenesis, imaging, histological, and immunohistochemical characteristics, treatment, and focus on the differential diagnosis of this rare tumor.

## Introduction

Metaplastic breast carcinoma (MBC) is a heterogeneous group of malignant epithelial tumors that undergoes metaplasia into mesenchymal or squamous differentiation. This group currently includes fibromatosis-like metaplastic carcinoma, low-grade adenosquamous carcinoma, spindle cell carcinoma, carcinoma with mesenchymal differentiation, and squamous cell carcinoma. They are very rare accounting for less than 1% of breast carcinomas. Each subtype has a unique morphology and different prognosis. Matrix-producing carcinoma (MPC) is a rare subtype of MBC. It is defined as a carcinoma with a direct transition from invasive carcinoma no special type to cartilage or osseous component lacking an intervening spindle cell component [1]. Because of rarity, the literature on MPC is limited and consists mostly of case reports and a few case series.

We present a case of MPC with excellent clinical outcome and focus on the differential diagnosis. We also discuss the histogenesis, clinical and imaging findings, histological and immunohistochemical profile, treatment, and prognosis of this rare entity.

## Case presentation

**Figure 1:**
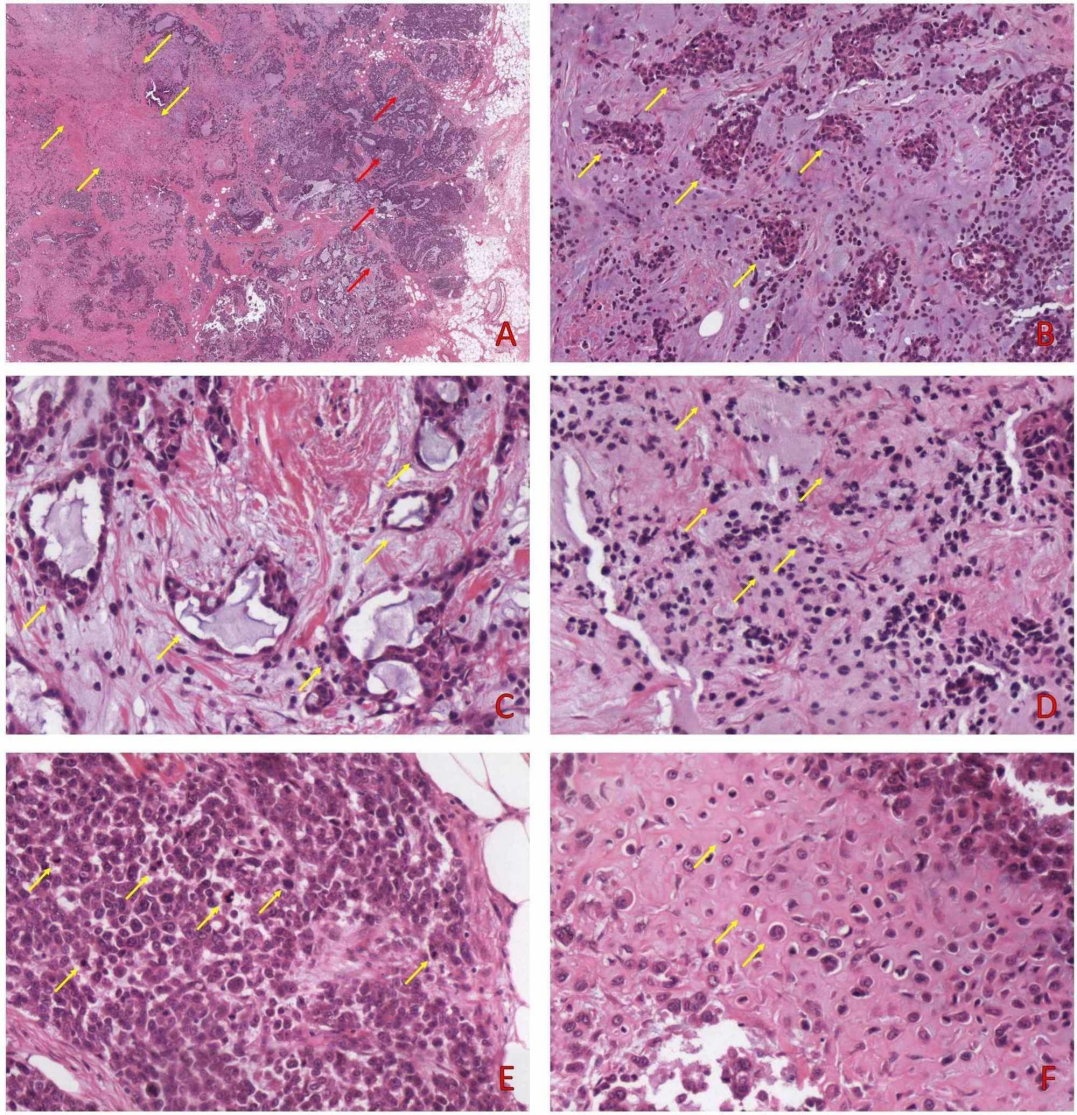
(A) On low power examination, MPC is more cellular peripherally (red arrows) with diminishing cellularity towards the center (yellow arrows); H&E x10, (B) nests, (C) tubular structures and (D) isolated tumor cells embedded in an extracellular myxoid matrix; H&E x200, (E) on high power examination, tumor cells were pleomorphic, had enlarged nuclei with distinct nucleoli. Several mitoses were identified; H&E x200, (F) the matrix cells were hyperchromatic, had irregular nuclear contour, and a distinct nucleolus was visible; H&E x200 H&E: hematoxylin and eosin; MPC: matrix-producing carcinoma.

A 65-year-old female patient with no previous history was admitted to the surgery department due to a palpable mass of the upper-outer quadrant of the left breast. A Breast Imaging Reporting and Data System 5 lesion measuring 23 mm in its greatest diameter were revealed on digital mammography. Frozen section was positive, while the sentinel lymph node biopsy was negative for malignancy. A mastectomy was performed (Poster presentation: Koufopoulos N, Pigadioti E, Dimas D, Tsouma E, Misitzis I, Khaldi L. Matrix Producing Breast Carcinoma. Report of a Case. 30th European Congress of Pathology; September 8-12, 2018). On gross examination, the tumor was relatively well-circumscribed, solid, and gray-white in color. On microscopic examination, the periphery of the tumor was more cellular with gradually diminishing cellularity towards the central area (Figure 1A). It consisted of solid areas, nests, tubular structures, as well as isolated single cells embedded in an extracellular chondromyxoid matrix (Figures 1B-1D). The matrix was diffuse accounting for 50% of the tumor area. Tumor cells had enlarged nuclei with distinct nucleoli (Figure 1E). The matrix was high-grade according to the criteria set by Downs-Kelly et al. (Figure 1F) [2]. Focal areas of necrosis were present. An intervening spindle cell component, angiolymphatic invasion, or peripheral lymphocytic infiltrations were not identified.

Immunohistochemical study was negative for Her-2, estrogen and progesterone receptors, cytokeratin 5/6, epidermal growth factor receptor (EGFR), P63, and S100. Proliferation index Ki67 stained 60% of tumor nuclei.

The final diagnosis was MPC of the breast. The patient received adjuvant chemotherapy and is alive without evidence of recurrence or distant metastasis 38 months after surgery.

## Discussion

MBC is a heterogeneous group of malignant epithelial tumors encompassing several different histologic subtypes, each one with a unique morphology and different clinical behavior. MPC is a rare subtype of MBC first reported in 1989 by Wargotz and Norris [1]. Its histogenesis is still unclear. Current literature suggests that MPC cells show epithelial features, either ductal or myoepithelial [3-4]. The neoplastic transformation of multipotent stem cells has been proposed in a few studies [5]. In some occasions, MPC has been associated with microglandular adenosis [6]. Schwartz et al. have recently reported a case of microglandular adenosis with evidence of molecular progression to MPC [7].

Clinically its presentation as a rapidly enlarging breast tumor is similar to other high-grade breast carcinomas of no special type [8]. On imaging studies (contrast-enhanced computed tomography and magnetic resonance imaging), MPC presents as a low-density mass with a peripheral ring structure enhancement [9-10]. On gross examination, the tumor is multinodular, well-circumscribed, hard in consistency, occasionally necrotic, and its color is grey-white. Microscopically, the tumor consists of malignant epithelial cells arranged in sheets, nests, cords, and tubular structures admixed with chondromyxoid or osseous matrix and sometimes with foci of hyaline cartilage [2]. Tumor cellularity is higher in the periphery and is gradually diminishing towards the center. Cellular atypia ranges from moderate to high-grade. Based on the nuclear characteristics (contour, pleomorphism, hyperchromasia), and whether visible nucleoli are present, the matrix has been classified as low-grade versus high-grade. Immunohistochemically, tumor cells usually express cytokeratin AE1/AE3, EGFR, and S100. They have almost always a triple-negative (ER, PR, and HER-2) immunophenotype.

MPC diagnosis is only possible in surgical specimens. Extensive sampling is required.

The differential diagnosis includes central acellular carcinoma (CAC) [11], primary or secondary chondrosarcoma, malignant phyllodes tumor with heterologous (chondorsarcomatous) differentiation [12], carcinoma ex-pleomorphic adenoma of the breast [13], and invasive lobular carcinoma with extracellular mucin production [14]. CAC and MPC both have a central acellular zone with a ring-like appearance. Their differences are that CAC lacks the chondromyxoid matrix of MPC and that there is a gradual decrease of neoplastic cells toward the central matrix in the MPC, whereas the transition between the cancerous and acellular zones in CAC is abrupt [15]. Proof of epithelial differentiation either morphologically or by immunochemistry excludes chondrosarcoma and malignant phyllodes tumor. Carcinoma ex-pleomorphic adenoma of the breast is composed of two components, one histologically similar to pleomorphic adenoma of the salivary gland and a malignant one resembling MPC [13]. In MPC the benign component is absent. Invasive lobular carcinoma with extracellular mucin production may also be included in the differential diagnosis since its mucinous component (single cells, clusters or cribriform structures floating in pools of mucin) may be confused with the matrix of MPC. However, the former shows non-mucinous areas of classic or solid type of invasive lobular carcinoma, signet ring cells, expression of estrogen receptors, and lack of E-cadherin expression [14].

MBC is usually treated aggressively due to the high stage at presentation [1]. Optimal treatment for MPC has not yet been defined since data are limited because of its rarity. Targeted gene therapy seems to play a role following genetic profiling in recent clinical trials [16].

Regarding prognosis, the literature has controversial results with reports claiming that MPC has a similar [1,3] or a more aggressive clinical behavior than invasive breast carcinoma of no special type [2]. Compared with other types of MBC, it has a better prognosis [1]. Large tumor size (more than 5 cm), lymph node infiltration, and high-grade matrix are associated with a worse outcome [5]. High-grade matrix is not related to tumor recurrence. Tumors with a significant amount of matrix (40% or more) seem to have a better prognosis [2].

In our case, we believe that the small tumor size, lack of lymph node infiltration, and the high percentage of the matrix may explain the lack of local recurrence or distant metastasis, despite the high-grade matrix.

## Conclusions

MPC is a rare subtype of MBC with unique morphology, imaging features, and a triple-negative immunophenotype. Accurate diagnosis is impossible in core needle biopsy specimens and requires a surgical sample. It is usually treated with surgery, followed by adjuvant therapy. However, due to its rarity, optimal treatment has not yet been defined. Its prognosis is better compared to other types of metaplastic breast carcinoma.
